# Navigating uncertainties for promoting nurse-led changes in work environments: A participatory action research

**DOI:** 10.1016/j.ijnsa.2024.100265

**Published:** 2024-11-12

**Authors:** Julia van Kraaij, Lotte Spruit-Bentvelzen, Famke van Lieshout, Hester Vermeulen, Catharina van Oostveen, RN2Blend consortium

**Affiliations:** a Radboud University Medical Center, IQ Health Science Department, Nijmegen, the Netherlands; b Erasmus MC, University Medical Center, Department of Quality and Patient Care, Rotterdam, the Netherlands; c Utrecht University of Applied Sciences, Utrecht, the Netherlands; d HAN University of Applied Sciences. Faculty of Health and Social Studies, Nijmegen, the Netherlands; e Spaarne Gasthuis Academy, Spaarne Gasthuis, Hoofddorp, the Netherlands; f Erasmus School of Health Policy & Management, Erasmus University, Rotterdam, the Netherlands

**Keywords:** Nurses, Nursing research, Uncertainty, Resilience, Action research, Work environment

## Abstract

**Background:**

The nursing work environment is crucial for nurses' well-being and patients’ quality of care. Despite effective interventions to improve the nursing work environment, understanding the most effective types and integration mechanisms for nurses remain challenging. As nursing practices evolve amid complex care demands and staff shortages, understanding nurses’ challenges, support systems, and adoption mechanisms is vital to optimize the work environment and to enhance quality of care, job satisfaction, and staff retention.

**Purpose:**

To explore strategies for promoting and supporting nurse-led changes to improving their work environment.

**Methods:**

The study employed a participatory action research design on three nursing wards in a Dutch academic hospital. Action research teams with diverse ward nurses were established on each ward. The research took place between September 2022 and October 2023. Data were collected during observations, PhotoVoice workshops, and individual interviews. Data were analyzed using the rigorous and accelerated data reduction technique.

**Results:**

Nurses and managers showed patterns of behavior that either hindered or facilitated changes, leading to the emergence of three themes: strengthening relationships, taking the lead, and being up to the task. These themes highlight the importance of fostering collaboration, encouraging proactive attitudes, and building capabilities to address challenges and drive positive changes in their work environment. Feelings of uncertainty emerged in all themes, and this uncertainty hindered nurses from taking responsibility for facilitating change.

**Conclusions:**

This study demonstrated different mechanisms that either facilitate or hinder nurse-led changes and how feelings of uncertainty play a role. Nurses emphasized the importance of collaboration and proactive attitudes but faced challenges in recognizing responsibility and their perceived competencies. Strengthening nurses' resilience to and management of uncertainty is essential. Healthcare organizations should help nurses navigate uncertainty to foster positive changes.

**Tweetable abstract:**

Collaboration, proactivity, and competency are key in nurse-led changes. Strengthening nurses' resilience and uncertainty management is crucial.

**What is already known**

• The nursing work environment significantly impacts both nurses and patients; positive environments are associated with better nurse wellbeing and quality of patient care.
• Various interventions demonstrate potential for improving the nursing work environment, yet uncertainty exists regarding their optimal efficacy.
• Nurses' engagement in change initiatives is crucial for creating a positive work environment, yet there is a gap in understanding the mechanisms that influence nurses’ ability to drive changes and integrate them into daily practice.

Alt-text: Unlabelled box

**What this paper adds**

• This study highlights the crucial interplay between uncertainty and responsibility in shaping nurses' ability to drive changes in their work environment.
• Strengthening relationships, fostering proactive attitudes, and enhancing competencies in nurses are crucial to stimulating resilience to uncertainty and to driving positive changes within their work environments.
• Participatory action research is valuable in stimulating nurses to enhance their own work environment by contributing to decision-making processes and collaborative problem-solving.

Alt-text: Unlabelled box

## 1 Introduction

The nursing work environment directly impacts the well-being of both nurses and patients. Nurses who work in supportive and well-organized environments are more likely to provide high-quality care, leading to lower mortality and adverse events (Nascimento & Jesus, 2020), while also experiencing greater job satisfaction and well-being, which is crucial for retaining nursing staff (Wei et al., 2018). Therefore, investing in improving the nursing work environment could significantly alleviate the challenges currently facing healthcare systems, including staff shortages and the growing complexity of care (Ball et al., 2023; Copanitsanou et al., 2017; Smith, 2018).

The nursing work environment is multifaceted and encompasses specific organizational characteristics such as culture, processes, and structures that impact professional nursing practice (Lake, 2002; Maassen et al., 2021). Over the years, numerous interventions have been developed to enhance the nursing work environment. Various reviews have outlined effective interventions, including those focusing on processes, psychosocial aspects, or digitalization (Eva et al., 2024; Paguio et al., 2020). These studies have demonstrated that interventions within the work environment can improve outcomes for nurses, patients, and organizations alike. However, although many interventions have proven effective, studies have primarily focused on quantitative results, and the most efficacious type of intervention remains uncertain (Paguio et al., 2020; Wei et al., 2018).

In effective interventions within nursing environments, key features include focusing on improving processes in the nursing work environment, employing participatory strategies, implementing changes at the unit level, and involving both frontline nurses and leaders (Paguio et al., 2020). Moreover, it is emphasized that nurses should take on the role of change agents (Wei et al., 2018). This highlights that nurses play a vital role in fostering and sustaining a positive work environment and that limiting their engagement in interventions risks valuable insights and perspectives being overlooked (Eva et al., 2024; Paguio et al., 2020; Wei et al., 2018). When nurses engage in change initiatives, they are encouraged not only to take ownership of their practice but also to foster (collective) leadership, facilitate professional growth, and potentially enhance a strong professional identity (Rasheed et al., 2020; van der Cingel & Brouwer, 2021).

Dutch nursing work environments are currently changing, with many hospitals transitioning towards differentiated nursing practices with varying competencies and education levels among nurses. By enabling nurses to leverage their expertise and skills, while fostering a diverse workforce that includes roles such as change agents, this approach has the potential to enhance the provision of high-quality care (Felder et al., 2024; van Kraaij et al., 2022). However, there are inherent complexities involved in facilitating these changes within the nursing work environment (van Kraaij et al., 2022). Hospitals face challenges as they navigate intricate practices influenced by historical and socio-political factors that shape nursing debates and practice (Schalkwijk et al., 2024). Previous research has taught us that we must involve nurses in such changes, yet the exact approach and effectiveness remain unclear. We have recognized the necessity of community-up approaches to mobilizing nurses and changing the work environment (van Kraaij et al., 2022), but a comprehensive understanding of nurses' ability to integrate these approaches into their work is imperative.

For these reasons, further research focusing on nurses' engagement in their work environment is necessary. We need insight into the challenges nurses face, the support they receive, and the factors that either facilitate or hinder their role in changing their work environment. To address this, this study explores strategies for promoting and supporting nurse-led changes aimed at improving their work environment. This understanding could facilitate the development and implementation of interventions to optimize the nursing work environment, potentially improving the quality of care and increasing job satisfaction and nurse retention (Ball et al., 2023; Copanitsanou et al., 2017; Smith, 2018; Wei et al., 2018).

## 2 Material and methods

### 2.1 Design

The participatory action research design was suitable for this study for several reasons. Firstly, involving nurses as active participants in the research helps them to contribute their insights, experiences, and suggestions for enhancing their work environment. Participatory action research actively involves nurses, helping them to develop a sense of ownership of their practice and commitment to facilitate changes (Kemmis et al., 2014; van Lieshout et al., 2021). This is important for achieving successful outcomes and ensuring dedication to change initiatives (Rasheed et al., 2020; van Kraaij et al., 2022).

Throughout our study, we followed a critical participatory action research approach, deeply rooted in the critical theory paradigm (McTaggart, Nixon, & Kemmis, 2017). This approach emphasizes an iterative process of reflection and action, where we as researchers were actively engaged in the practice under study. We continuously reflected, often also with participants, on various elements such as positions, norms, interests, and our own assumptions. This ongoing reflection enabled us to gain deep insights into the work processes and to develop knowledge within the specific context of the practice. Such insights are essential for formulating effective and enduring strategies for improvement (Kemmis et al., 2014; van Lieshout et al., 2021).

### 2.2 Context and participants

The participatory action research was conducted on three wards (Table 1) in a Dutch academic hospital between September 2022 and October 2023. Wards were selected based on the following factors: (1) authorization to conduct the research from cluster managers; (2) an intention to introduce differentiated nursing practice; and (3) the inclusion of diverse specialisms. On each ward, an action research team with clinical ward nurses was established. Nurses could express their interest in joining the action research team after attending information sessions within their respective wards, and unit managers directly invited select nurses to (voluntarily) participate. These nurses were selected based on their potential valuable contribution to the action research team, either personally or professionally, or to ensure representation of the ward. The action research teams encompassed diverse levels of experience and educational backgrounds, including vocational and bachelor's degrees. All action research team nurses were clinical nurses, and some were senior nurses. Senior nurses are responsible for coordinating and overseeing patient care within the wards, ensuring that the needs of both patients and nursing staff are addressed. When forming the action research team, differentiated nursing practice was also considered by introducing the ‘nurse coordinator’. These are nurses with a bachelor-level competency who lead nursing care for patients and families within a care pathway that focuses on innovation, professional development, coaching, evidence-based practice, and improving care quality (Personal communication, July 1, 2022). Each action research team included at least one aspiring nurse coordinator. During the study period, one ward initiated the implementation of nurse coordinators. All action research team nurses were allowed to allocate approximately two hours per week to this research. The unit managers engaged in the participatory action research and were informed of the progress, but allowed the action research team nurses take the lead. One nurse from each action research team withdrew their participation, citing reasons including securing a new job and being too busy with other responsibilities.

**Table 1:** Description of participating wards

Ward	Specialism	Number of beds	Number of registered nurses (% bachelor trained)	Number of certified nursing assistants/health and welfare assistants	Action research team nurses
Ward A	Medical unit	32	50 (40%)	7	12
Ward B	Medical-surgical unit	39	40 (40%)	3	6
Ward C	Medical unit	56	72 (55%)	0	5

### 2.3 Data collection

**Fig. 1:**
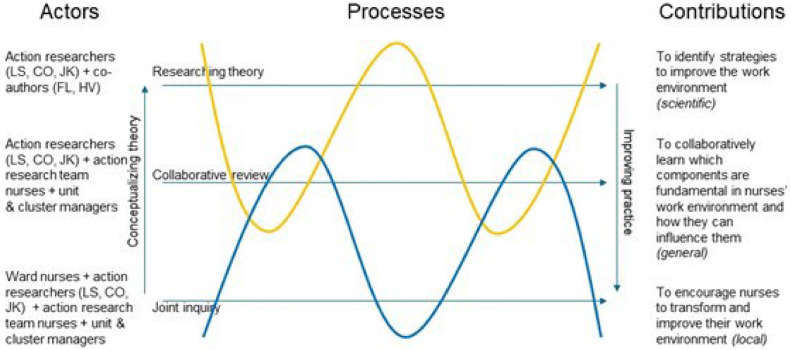
The research design and process intertwine three key processes: researching theory, collaborative review, and joint inquiry. The lines within the design visually represent the stages of this process, illustrating how all elements are interconnected and flow together to inform and shape the overall research process (adapted from Schuiling and Kiewiet (2016)).

An important characteristic of participatory action research is the continuous balancing act between developing theory and improving practice. Our study design was based on the Triple Process Structure model by Schuiling and Kiewiet (2016) (Fig. 1). This model integrates theory and practice and distinguishes contributions at three levels: the local practice contribution, the general practice contribution, and the scientific body of knowledge. This paper further explored the goal of scientific knowledge, complementing the focus of the first two contributions addressed during the practical research.

While consistently navigating the three processes outlined by Schuiling and Kiewiet (2016), we structured our data collection into four phases (Table 2)*: pre-orientation, orientation, planning and testing actions, and evaluation* (Kemmis et al., 2014; van Lieshout et al., 2021). These phases helped us to consistently navigate the outlined processes while addressing the three formulated contributions.

**Table 2:** Overview of research phases, goals, data collection activities, and time in hours (see Supplementary File 1 for a specification of the activities and time in hours)

Phase	Goal	Activities	Time in hours
*All phases*	To facilitate collaboration, communication, and discussion among researchers and action research team nurses, providing a platform for sharing insights, and collectively making decisions related to the research or intervention	Action research team meetings	50 (38 meetings)
Pre-orientation	To obtain approval, to inform, to determine research focus, and to recruit participants for the action research team	Pre-discussions and alignment of the research with cluster and unit managers	3 (3 sessions)
	To provide information about the research	Information sessions with ward nurses	7 (7 sessions)
Orientation	To explore the current situation	Observations on three wards	52 (13 observations)
	To explore the current and desired situation	Photovoice	8 (6 sessions)
	To explore the current and desired situation	Semi-structured interviews	8 (16 interviews)
	To give an update on the research project and share the findings and potential actions	Meetings with cluster managers	1 (2 sessions)
Planning and testing actions	To implement and execute the formulated actions on the wards	Team workshops	7 (3 sessions)
Evaluation	To systematically evaluate the research	Meetings with action research team nurses and unit managers	3 (3 sessions)
	To provide information about the executed activities	Information session with ward nurses	1 (1 session)

Action research team meetings took place throughout the participatory action research to ensure theory and practice were integrated (process of collaborative review according to Schuiling and Kiewiet (2016)). These meetings served as a platform for sharing insights, reviewing progress, addressing challenges, and collectively making decisions related to the research or intervention being carried out. We continuously evaluated our discoveries, examining literature to identify successful and unsuccessful approaches and exploring which interventions proved effective.

#### 2.3.1 Pre-orientation

Relationship building was the main activity during this phase. We informed and obtained final approval from the management, briefed the participating nursing wards, invited nurses for the action research teams, and determined the research focus for each ward. To guide our focus, we utilized an annual survey assessing the quality of nurses' work environment, which included the Practice Environment Scale of the Nursing Work Index (Lake, 2002). This survey was conducted locally at the hospital's initiative to assess the quality of nursing care and evaluate its organizational aspects. We selectively incorporated findings relevant to the three wards involved in our present study. Using these results, we engaged in discussions with ward nurses during plenary sessions to identify important themes related to transforming their work environment. Multiple chances for improvement were acknowledged in this context, and three key challenges were identified on the three wards. Ward A, characterized by numerous medical specialties and a high personnel turnover, faced challenges in staying updated on medical content, with little room for extra training and improvement. In Ward B, which had a stable team and efficient working processes, there were difficulties involving the team in the implementation of protocols or execution of new work procedures. Ward C, known for its individualistic way of working and highly protocolized work procedures, was a place where nurses desired mutual involvement and connection during their work.

#### 2.3.2 Orientation

During this phase, building upon the knowledge gained in the previous phase, we aimed to better understand the work environments and identify opportunities for improvement. This was achieved through observations, PhotoVoice workshops with the nursing teams, and individual interviews. Observations gave us a realistic view of the current situation and allowed us to give immediate feedback to the nurses. Prior to the PhotoVoice sessions, we asked nurses to take two pictures of specific aspects in their work environment: one they considered positive and one negative. During the sessions, we facilitated reflection and discussion using the SHOWeD technique, which comprised five questions (Versey, 2024).

• what do you see here?
• what's really happening here?
• how does this relate to our (work)lives?
• why does this problem, concern, or strength exist?
• what can we do about it?

The researchers took field notes during these sessions. Directly after the observations and PhotoVoice sessions, semi-structured interviews were conducted with ward nurses to discuss and reflect on our observations. In total, 16 interviews were conducted with ward nurses selected by purposive sampling. The action research team nurses approached colleagues with a variety of ages, backgrounds, and work perspectives to participate in an interview. Nurses who agreed to participate were invited to schedule an interview (face-to-face or online using Microsoft Teams). For each ward, a different interview guide was constructed depending on the research focus (Supplementary file 2). Each interview was audio recorded and transcribed verbatim.

#### 2.3.3 Planning and testing actions

This phase focused on the development and testing of a chosen intervention in practice. Ward A did not proceed to this phase. In Ward B, the emphasis was on implementing interventions that either facilitated or impeded changes. For instance, we conducted team meetings with exercises to embrace change and improvisation workshops. In Ward C, we established a buddy system in which evaluation moments were converted into opportunities for reflection. We discussed progress during the action research team meetings by sharing the experiences with testing the interventions and making sense of them collectively.

#### 2.3.4 Evaluation

The research project was systematically evaluated with the action research teams during this phase. A reflection meeting was organized for nurses and unit managers, where we collected their thoughts on the role of the action research team, individual contributions, and our role as researchers in the project. We also gathered insights and advice on designing future research. The discussions also considered how the action research teams could sustain the intervention and approach future changes. While the action research teams continued with the project themselves, our role as researchers concluded after these evaluation sessions.

### 2.4 Data analysis

Data were collected and analyzed in parallel processes, as analysis occurred gradually throughout the different action research phases. Collaborative analysis took place during meetings, observations, and interviews, providing a foundation for reflecting on patterns, underlying causes, and themes with the researchers, action research team nurses, and unit managers (Cornish et al., 2023). We also used the rigorous and accelerated data reduction technique for comprehensive analysis. This entails a systematic and incremental approach to reduce data and prioritize essential information, and consists of five steps (Watkins, 2017). In the first step, we verified consistent formatting by keeping a logbook of data collection, including references to minutes, transcripts, or audio files. In the second step, we created a data reduction table using Microsoft Excel. In this table, raw data were included and divided over the three wards. Two researchers (LS, CO) reviewed the table in line with the aim of the study and research question (*what strategies can be employed to promote and support nurse-led changes aimed at enhancing the nursing work environment?*). In the third step, we reduced information that did not appear of interest for the analysis and subcategorized the data into 55 codes. Subsequently, semi-final decisions were made about inclusion of codes. In the fourth step, LS, CO, and JK independently went through this data table and categorized the codes into six overarching themes: ownership, skills, collaboration, learning and reflection, management support, and leadership. In the fifth step, the researcher not engaged in the coding process (FL) peer reviewed the themes. All authors engaged in critical discussions about the recognized themes and grouped those themes that overlapped. Four overarching themes and 28 underlying codes were identified. With these themes and codes, we revisited the raw data and supplemented the themes with quotes, ensuring accuracy and completeness, with independent oversight from three authors (JK, LS, CO) (Supplementary File 3). We structured the result section around three themes, as we have integrated the theme ‘support from manager’ into the others.

### 2.5 Rigor and trustworthiness

Participatory action research carries moral and political weight as it aims to address power dynamics and imbalances between stakeholders, while also generating valuable knowledge for a wider audience (Kemmis et al., 2014). The main premise of this participatory action research was setting goals for local and general practice and generating (scientific) knowledge (Schuiling & Kiewiet, 2016). We also employed an iterative process, continuously refining strategies and actions through collaborative reflection and feedback. This helped us identify potential biases in data interpretation, while considering participation dynamics, relationships, and potential consequences of our actions (Kemmis et al., 2014). The reflective and thorough discussions between the researchers, action research team nurses, and unit managers, enhanced the credibility and confirmability of this research. All authors are non-practicing registered nurses. JK is a nursing science PhD candidate and holds MSc degrees in business administration and health and life sciences. LS holds a MSc degree in nursing science and is a nursing staff advisor. FL is an associate professor with expertise in participatory action research and development of effective workplace cultures. HV is a professor of nursing science and clinical epidemiologist. CO is a nursing dean and senior researcher. All authors participated in analyzing and interpreting the findings and finalizing the article. We used the Standards for Reporting Qualitative Research guidelines to ensure consistency and rigor throughout the research process (O'Brien et al., 2014).

### 2.6 Ethical considerations

The study was approved by the local medical ethics review board of the Radboud University Medical Center (study number 2019-5992), and the need for ethical approval for human subject research was waived. Written consent was obtained from all ward nurses, emphasizing voluntary participation, confidentiality, and anonymity. Ward nurses were informed of their right to withdraw at any time. We also adhered to the fundamental principles of research ethics by respecting all individuals involved and honoring their integrity (Kemmis et al., 2014). Data were anonymized and stored according to the regulations of the Radboud Academic Medical Center.

## 3 Results

We explored the conditions influencing nurses' capability to foster ownership, strategies to encourage nurses’ responsibility, and willingness of nurses to change their work environment. Although the wards were diverse and faced different challenges, making a change in the work environment appeared to be a big challenge for all wards. We identified patterns in the behavior of nurses and their managers that either hindered or facilitated changes. Three overall key themes emerged: (1) strengthening relationships: fostering a sense of community and collaboration; (2) taking the lead in improvement and change: the necessity for a proactive attitude; and (3) being up to the task: the need for perceived competence. Nurses faced uncertainties within these themes, hindering their ability to take responsibility.

### 3.1 Strengthening relationships: fostering a sense of community and collaboration

The development of sense of community and collaboration among ward nurses appeared to be an important aspect when changing the work environment. We observed two forms of collaboration among nurses: (1) social interactions and a sense of belonging during work and (2) collaborative problem-solving.

During the PhotoVoice sessions on all three wards, nurses captured photos depicting a good atmosphere and appreciation from colleagues. These images included positive messages exchanged in the coffee room and treats from both colleagues and patients: *“Having put in your utmost effort for three weeks straight, appreciation is truly welcomed. Delightful treats then help to turn things around”* (9/11, PhotoVoice, nurse, ward B).

Engaging with each other during work was identified as an important aspect: *“We have a nice team and nice colleagues, we support each other; this influences the quality of care. Patients also appreciate and experience this”* (9/11, PhotoVoice, nurse, ward B). However, nurses did not always think there was enough of this: “*Due to the hard work, we don't always have time for each other or even to take care of ourselves”* (16/12, PhotoVoice, nurse, ward C). On one ward, it was not possible for the ward nurses to regularly interact or communicate with each other, despite its importance for fostering collaboration, because of the large team and rotating shifts: *“You don't know what's going on with everyone, because it's a big team”* (1/2, interview 6, nurse, ward C). This lack of mutual engagement surfaced during the scheduling process as colleagues frequently did not volunteer to exchange shifts. Moreover, three team managers were present on the largest ward, so establishing connections between nurses was even more complex. Engagement was more pronounced on one ward where managers actively invested in team-building activities.

Recognizing the importance of interaction, ward nurses acknowledged that providing feedback to address behavioral issues or commitments was easier when they had established relationships with their nurse colleagues: *“It is easier to address each other if you know each other better”* (24/2, interview 10, nurse, ward C). This quote emphasizes the importance of building strong interpersonal connections to facilitate effective feedback mechanisms and address issues proactively.

The need for collaborative problem-solving became apparent during the action research team meetings. This included teamwork, communication, and a shared commitment to addressing challenges. The ability to collaborate on a project, particularly when dealing with new and unfamiliar tasks, was recognized as crucial. *"When researching literature, it's nice to have a buddy instead of being alone, because there is less experience in this"* (26/5, evaluation, action research team nurse, ward A).

Action research team nurses were looking for an effective way to communicate within the group. They knew this was important, but it was challenging to keep everyone informed and engaged. *“The attendees took up the tasks and that made the ones that were not present less involved, because they did not know what to do”* (26/5, evaluation, action research team nurse, ward A).

### 3.2 Taking the lead in improvement and change: the necessity for a proactive attitude

The dedication of the ward nurses to the well-being of their patients drove them to take the lead when making clinical judgments and actions. For instance, we observed that ward nurses ensured a smooth progress of examinations, made sure that necessary checks were performed on time, and coordinated their schedules with physicians. *"I always schedule an early shift the day before the medical grand rounds to be adequately prepared. With just the handover and a quick overview, you do not have enough information"* (17/11, observation, nurse, ward B)

However, this seemed to be limited to patient care and ward nurses appeared somewhat negligent when asked about, for example, quality improvement projects, departmental change initiatives, or evidence-based practice. As one nurse mentioned: *“The team disengages and is difficult to reach, but for the patient, they do everything”* (14/12, action research team nurse, ward C). Despite this, some ward nurses recognized their important role: “*I think it is positive when nurses take the lead in changes. We are also at the bedside, and if we have to change things, it would be nice if we determine the bottlenecks ourselves. It can be determined from above, but then it [the solutions] is not always feasible.”* (19/1, Interview 2, Nurse, Ward B).

Managers also played a crucial role in helping nurses to embrace their roles and responsibilities, which included the provision of resources, encouragement, and empowerment to enhance their effectiveness. For example, the introduction of the role of a nursing researcher posed a significant challenge on one ward, as revealed in an interview: *“When I started my master education, I approached my managers to ask how we could structure or implement it. I asked, ‘Do you have any use for me?’ Well, that was all quite challenging, simply because it's unfamiliar territory. So, I didn't feel much cooperation there, and I still don't always”* (17/3, interview 11, nurse, ward A).

Ward nurses were able to come up with solutions to problems they experienced, but quickly dismissed these ideas because they did not consider it their task or responsibility to find solutions. For instance, during one of the PhotoVoice sessions, nurses mentioned that they faced challenges with tangled cords on electrical devices:

*"Before we can use the device, we spend a considerable amount of time untangling the cords. […] We use this device multiple times a day, and a better design would help us use it more effectively" (9/11, nurse, PhotoVoice, ward B).*

Despite having valuable ideas for improvement, none of the ward nurses submitted their proposals to the hospital's design improvement department, citing reasons like heavy workloads, lack of time, or a preference for on-site submissions. Nurses attributed the problems to external factors and felt a sense of resignation regarding their limited influence. Nevertheless, during an interview, it came to light that nurses might hastily conclude they lack influence when, in fact, they may be uncertain about whom to approach or about the appropriate course of action in such situations:

*“During the PhotoVoice sessions, I noticed remarks like, ‘I'm not happy about this, but I have no influence over it.’ However, I believe that, in some cases, these were matters where nurses could exert influence if they knew which routes to explore or with whom to engage in conversation”* (17/3, interview 11, nurse, ward A).

At the beginning of this project, the action research team nurses were uncertain about the project's timeline and about what was expected in terms of their roles and tasks. During the action research team meetings, we noticed that, when there was uncertainty, the tendency was often to not perform the task rather than seek help. The action research team nurses had difficulties with taking responsibility to clarify and address the problem: *"I often thought it was my fault if I didn't understand something. That hindered me from asking"* (26/5, evaluation, action research team nurse, ward A).

The action research team nurses were not accustomed to the level of responsibility assigned to them, as they typically received directions from their managers: *“Nurses do not determine policy, but participate in its execution”* (10/10, evaluation, action research team nurse, ward C). This observation became apparent in the action research team meetings, where nurses exhibited a tendency to turn to the manager expectantly when asked something. It turned out to be crucial for managers to possess the right competencies to motivate and encourage nurses to take responsibility, as one of the ward nurses mentioned: *“I think they are the biggest incentive to make us feel professional. They certainly play a role, both in organizing days for quality work and in strengthening our professional sense”* (17/3, interview 11, nurse, ward A).

The ability to deal with setbacks and seek help when needed emerged as important factors when taking control. For example, during one of the team meetings, action research team nurses encountered significant resistance from colleagues after presenting initial results of the project and potential solutions. A few ward nurses disagreed, leading to threats of resignation and personal feelings of attack among the action research team nurses. However, the action research team nurses perceived this as a window of opportunity for change and embraced the challenge of still getting the team on board with the proposed changes:

*“The team meeting was a turning point. Then we thought, okay, we really have to keep it much closer to the team, but we also really have to play our role in that”* (10/10, evaluation, action research team senior nurse, ward C).

When the action research team nurses assumed control, we noticed a notable improvement in the project's progress, enabling them to effectively initiate the intervention phase. This positive development was also recognized by the nurses themselves, as expressed during a project evaluation: *“Towards the end of the project, I got the idea that we had taken more control ourselves, and I think we learned a lot from that"* (10/10, evaluation, action research team senior nurse, ward C).

### 3.3 Being up to the task: the need for perceived competence

Taking on the responsibility to initiate changes seemed to be connected to the perceived level of competence to assume that responsibility. We observed that ward nurses showed greater readiness to take on responsibilities when they felt assured of their necessary skills. Initiating a shift with a critically ill patient was not stressful for a ward nurse who confidently stated: *“I've been in the business for a while. I have already seen and experienced a lot. This combination of theoretical knowledge and experience makes me flexible and agile. I adapt and I see what needs to be done”* (7/4, interview 14, nurse, ward A).

Ward nurses were very adept at recognizing and articulating challenges in their work environment. However, our observation revealed that they lacked the necessary skills to prioritize these challenges effectively. For instance, during a PhotoVoice session, ward nurses extensively discussed issues like disorderly wards and the absence of materials, which were essentially straightforward problems. There was no focus on addressing deeper structural or systemic challenges associated with these issues.

We became aware that, in the daily routine, a cyclical learning and improvement process was lacking among the nurses. Their solution-oriented approach was often to find temporary fixes and shortcuts instead of permanent solutions to enhance their work environment: *“Are we going to invest our energy in untangling the wires of electrocardiogram machines? I think it's a waste of my time.”* (9/11, PhotoVoice, Nurse, Ward B)

Moreover, ward nurses pointed out that the nursing process had evolved into a sequence of tasks facilitated by the highly task-oriented electronic patient record. This, coupled with nurses' tendency to focus on the immediate allocation of duties and patient care workload during evaluations, hindered the exploration of structural and systemic solutions. *“The point is that it is solved quickly, not that it is solved with good quality. I think that a structural approach, for something that works in the longer term, often fails to occur”* (17/3, interview 11, nurse, ward A).

The action research team initially led the project but encountered challenges as they grappled with how to approach and initiate it. We noticed that action research team nurses did not consider themselves skilled in the field of project and change management and evidence-based practice. One action research team nurse mentioned: “*I've had an afternoon course of project management, but I find it challenging to link that to this project”* (10/10, evaluation, action research team senior nurse, ward C).

While managers initially adhered to the request to step back and let action research team nurses lead the project, it became clear that ongoing managerial support was essential when nurses faced challenges initiating the project and assuming responsibility. Successful support included managerial guidance while delegating decisions regarding the content to the project teams: *"From the halfway point onwards we also had her [the manager] more as a source of information and then we just started sparring with her and you learned things from that*" (10/10, evaluation, action research team nurse, ward C). The research project stagnated in wards where this did not take place.

Positive impacts were also observed when managers engaged in self-reflection and encouraged a reflective approach. Action research team nurses emphasized the importance of being able to depend on their manager during challenging situations, appreciating a collaborative and supportive dynamic: *“Because as a manager I was expected not to direct anything, so I didn't do that. That also asked something of me. But at a certain point I intervened and said: ‘I think we now have to provide a little more guidance and give the working group more guidance about their role, the role of the researchers and the expectations’”* (10/10, evaluation, manager, ward C).

With managerial coaching and practical support, such as offering relevant literature and demonstrating procedures, action research team nurses grew confident in their own abilities. This increased perceived competence made them more proactive, wherein action research team nurses asked for help, talked about responsibility, and they explored possibilities: *“At the beginning, I did not really know what to do, but as I got further into the process I just knew what was expected of me, and did that, and I really enjoyed seeing that I got better and better at it”* (10/10, evaluation, action research team senior nurse, ward C). They also began to value the change process in their ward: “*I have come to appreciate the process of change more, instead of the result. Because the process determines success."* (6/7, Evaluation, action research team nurse, Ward B).

## 4 Discussion

The aim of this study was to explore strategies and mechanisms that promote and support nurse-led changes within the nursing work environment through participatory action research. We showed that feelings of uncertainty and responsibility affected nurses' ability to foster ownership and initiate changes in their work environment. We noted this pattern across three primary themes: (1) strengthening relationships: fostering a sense of community and collaboration; (2) taking the lead in improvement and change: the necessity for a proactive attitude; and (3) being up to the task: the need for perceived competence.

The first key finding was the importance of strengthening relationships in creating a positive work environment. We observed this as a need for community, collaboration, and teamwork among ward nurses. In line with earlier research findings (Ahlstedt et al., 2019; Hanafin et al., 2022), nurses were more likely to commit to and engage in their work and the organization when they felt connected to their colleagues and the organization. Managers played an important role here by promoting positive relationships between colleagues (Ahlstedt et al., 2019; Hanafin et al., 2022). We also noted the significance of collaborative problem-solving and fostering a supportive, collegial environment among nurses, which has been emphasized in previous research (Hörberg et al., 2023). Nurses showed a preference for teamwork, especially when dealing with uncertainties in task identification or execution, such as when they were assigned unfamiliar activities.

The second theme that emerged was the initiative to lead improvement and change within the work environment. We noticed that nurses were often primarily task focused and seemed to overlook their potential responsibility for initiating improvement and change. However, once they recognized their responsibility, they showed proactive mindsets. Pursio et al. (2021) defined nurses' participation in decision-making processes and their ability to influence them as *professional autonomy.* They underscored the importance of shared leadership and supportive nurse managers in empowering nurses to exert their influence. An open attitude towards change is (partly) fostered by managers being reliable, inspiring, and encouraging (Cheraghi et al., 2023). Nurses possessed a degree of influence over their work environment, yet they also relied on support from their managers. We demonstrated the important role of nurse managers in stimulating nurses to assert their (organizing) roles and encouraging them to construct solutions to barriers in their environment. This was illustrated in one action research team, which faced resistance from the nursing team on suggested interventions to improve the work environment. In this case, the unit manager successfully intervened by prioritizing the process over the content and by encouraging action research team nurses to take responsibility for their professional role. We also observed that uncertainties hindered nurses’ ability to initiate changes and that nurses mainly felt responsible for direct patient care rather than for initiating change. This can be attributed to the professional identity of nurses (Philippa et al., 2021; van der Cingel & Brouwer, 2021) and to deeply rooted organizational processes and structures tailored to bedside nursing work (van Kraaij et al., 2024, Felder et al., 2024). Nurses cannot take responsibility without adequate authorization, and organizational constraints such as unclear rules, hierarchical structures, and limited control over practice can impede this (Pursio et al., 2021). In addition, perceptions of the professional image and corresponding practices in nursing are not always contemporary. Interestingly, prior research attributed uncertainty in nursing practice to ambiguity within the nursing domain (Vaismoradi et al., 2011). Nursing is frequently seen as a profession dominated by women, with its professional identity influenced by subjective factors such as societal perceptions and stereotypes, rather than by the complex and professional roles and responsibilities it encompasses (Philippa et al., 2021; Teresa-Morales et al., 2022).

Nurses demonstrated proficiency in patient care; however, they perceived a deficiency in the skills necessary for prioritizing challenges and implementing structural and systemic improvements (van Oostveen et al., 2015). Due to time constraints, nurses often resort to working within established routines, neglecting to address the root causes of issues, and primarily engaging in first-order problem solving (Tucker & Edmondson, 2002).

Our final theme was the need for perceived competence to assume responsibility to improve the work environment. Nurses were proficient in patient care, but they perceived deficiency in the skills necessary to prioritize challenges and implement structural and systemic improvements (van Oostveen et al., 2015). Nurses had limited time for reflection, so frequently resorted to working in a routine manner, without addressing underlying causes of problems and essentially engaging in first-order problem solving (Tucker & Edmondson, 2002). In this context, nurses should prioritize supporting each other in learning and reflection over assisting one another in completing patient care. To foster a culture of continuous improvement among nurses, a shift towards a second-order learning approach involving cyclical learning and improvement is needed (Tucker and Edmondson, 2002). Earlier studies have demonstrated that differentiated nursing practice could help nurses use this second-order approach to address issues (van Kraaij et al., 2024). However, this entails more than just altering roles and job descriptions; it requires a complete organizational transformation that includes nurses. Organizations must establish a culture of mutual accountability for implementing solutions and efforts should focus on building confidence among employees that their contributions matter (Mazur et al., 2012). Aligning processes, structures, and relationships across the organization is essential to support second-order learning behaviors among nursing teams (van Kraaij et al., 2022; van Schothorst-van Roekel et al., 2020). This alignment fosters an environment where nurses engage in cyclical learning and improvement, promoting ongoing enhancements in healthcare delivery.

These three themes are interconnected, and uncertainties have hindered nurses’ ability to take on responsibilities. To assume responsibility effectively, nurses need to have the necessary competencies, foster effective collaboration, and receive strong managerial support, which includes relational support and an unconditional trust and belief in nurses’ capacity to act. This enables them to navigate uncertainty and take control of their responsibilities confidently. Research on uncertainty in nursing practice is sparse and has mainly focused on uncertainty in clinical practice (Vaismoradi et al., 2011). However, an earlier study on work environment uncertainty and organizational readiness for change among nurses showed that knowledge, skills, and aptitudes of nurses need to be promoted so they can respond to uncertainty (Alkarani, 2023). The consistency of these themes corresponds with the principles of self-determination theory, which underscores relatedness, highlighting the importance of belonging, feeling connected with others, autonomy, self-direction, and a sense of independence and competence. This stresses the significance of feeling effective and capable. These factors are essential in fostering intrinsic motivation, well-being, and organizational effectiveness (Deci et al., 2017; Ryan & Deci, 2000). Establishing a healthy work environment requires joint effort from nurses, managers, and the organization. When nurses perceive a supportive and trustworthy environment, they are more likely to feel encouraged and motivated to confront uncertain situations and eventually make improvements in their work environment.

### 4.1 Strengths and limitations

A strength of this study is that participatory action research enabled nurses to give their perspectives and experiences on the mechanisms that either facilitate or hinder nurse-led changes (Kemmis et al., 2014; van Lieshout et al., 2021). Our participatory approach not only fostered learning and reflection but also encouraged nurses to engage in second-order problem-solving and lead changes themselves. With our guidance and support, they learnt how to adjust or implement changes. This process of learning and change allowed us to explore mechanisms and acquire evidence-based insights into strategies and their contributions to facilitating nurse-led changes that improve the work environment. This underscores the potential of participatory action research in enabling nurses to take responsibility and to actively contribute to positive changes. To our knowledge, this is the first study to investigate how these mechanisms operate in practice and to identify what nurses require to change their work environment. Potential limitations of participatory action research are challenges such as social desirability bias and potential conflicts of interest. To address these issues, we maintained a critical attitude and practiced reflexivity to interpret nurses' experiences and minimize bias (Bispo Júnior, 2022). Another potential limitation is that we did not use a participatory approach with the action research team nurses to code the data. However, data were analyzed iteratively throughout the research after various activities. Findings were discussed with the action research team nurses, and new data collection activities were planned based on these analyses. Although this study was conducted in one Dutch academic hospital, we believe that our findings and implications can be applied to other healthcare settings. A further limitation is that the study was not long enough to fully uncover long-term impacts. However, the focus was on processes within different action research teams and the strategies used to activate them. We observed similar patterns on the wards, but ongoing evaluation and adjusting strategies at the ward level will be crucial to ensure sustained improvements.

### 4.2 Implications

These findings have several implications for nursing practice and research in improving the nursing work environment. We have shown that raising awareness about the fundamental motivational needs of strengthening relationships, fostering proactive attitudes, and enhancing competencies can reveal nurses’ motivations for change (Ahlstedt et al., 2020). Improving nurses' resilience and ability to handle uncertainty can facilitate successful transitions in the work environment (Hörberg et al., 2023). Developing skills in uncertainty management among nurses could further foster a supportive and collegial environment, the assertion of autonomy, and the development of perceived competence. These skills include developing personal reflection skills (Hörberg et al., 2023) and second-order problem solving skills (Tucker & Edmondson, 2002). Routine first-order problem solving restricts the implementation of structural changes, which has implications for both education and practice. Alongside regular nursing training, which predominantly focuses on clinical situations, courses should equip nurses with strategies to navigate uncertainty (Hörberg et al., 2023). We also highlighted that nursing managers play a crucial role in promoting and supporting resilience among nurses by encouraging involvement, fostering positive relationships, acknowledging nurse responsibility, investing in competency development, and creating a supportive environment where nurses feel valued, respected, and empowered to voice their concerns and ideas (Cheraghi et al., 2023; Morrison & Jensen, 2022). Further research could investigate the long-term effects of participatory action research interventions on nursing environments and the continuous evaluation of strategies at ward levels to sustain improvements. Future work should also explore how nurses manage uncertainty as these skills have not been well studied, particularly regarding how they affect quality improvement and change initiatives.

## 5 Conclusions

This study has contributed to the sparse research on strategies for promoting nurse-led changes through participatory action research. The interplay between responsibility and uncertainty influenced nurses' ability to initiate changes within their work environment. Nurses emphasized collaboration and proactive attitudes in addressing uncertainties but faced challenges in recognizing their responsibility and their perceived competence for initiating structural changes. Strengthening nurses' resilience and uncertainty management skills through reflective practices and proactive problem-solving is crucial for successful transitions in the work environment. Hence, healthcare organizations should raise awareness of nurses' fundamental motivational needs and support them in navigating uncertainty to foster positive changes.

## Funding sources

This work was supported by Dutch Ministry of Health, Welfare, and Sports (project number 1532566-190809-MEVA).

## Declaration of generative AI in scientific writing

During the preparation of this work, the authors utilized ChatGPT to enhance the readability and language of the manuscript they originally wrote. After using this tool, the authors reviewed and edited the content as needed and take full responsibility for the final content of the published article.

## CRediT authorship contribution statement

**Julia van Kraaij:** . **Lotte Spruit-Bentvelzen:** Writing – review & editing, Investigation, Formal analysis, Conceptualization. **Famke van Lieshout:** Writing – review & editing, Methodology. **Hester Vermeulen:** Writing – review & editing, Supervision. **Catharina van Oostveen:** Writing – review & editing, Supervision, Methodology, Investigation, Formal analysis, Conceptualization.

## Declaration of competing interest

The authors declare that they have no known competing financial interests or personal relationships that could have appeared to influence the work reported in this paper.

## Appendix B Supplementary materials

Image, application 1

Image, application 2

Image, application 3
