# Supplementary material for: Navigating uncertainties for promoting nurse-led changes in work environments: A participatory action research
Source: Int J Nurs Stud Adv. 2024 Nov 12;7:100265. doi: 10.1016/j.ijnsa.2024.100265 (PMC11625307; doi:10.1016/j.ijnsa.2024.100265)
Supplement: Supplementary file 1 [file mmc1.docx]

# Supplementary file 1. Data collection activities and time in hours

| Date | Phase^a^ | Activity | Ward | Time in h |
| --- | --- | --- | --- | --- |
| 27-5-2022 | 1 | Pre-discussions and alignment of the research with cluster and unit managers |  | 1 |
| 16-8-2022 | 1 | Information session with nurses to provide information about the research | B | 1 |
| 16-8-2022 | 1 | Pre-discussions and alignment of the research with cluster and unit managers |  | 1 |
| 19-8-2022 | 1 | Pre-discussions and alignment of the research with cluster and unit managers |  | 1 |
| 19-8-2022 | 1 | Information session with nurses to provide information about the research | C | 1 |
| 6-9-2022 | 1 | Information session with nurses to provide information about the research | C | 1 |
| 20-9-2022 | 1 | Information session with nurses to provide information about the research | A | 1 |
| 20-9-2022 | 1 | Information session with nurses to provide information about the research | B | 1 |
| 27-9-2022 | 1 | Information session with nurses to provide information about the research | A | 1 |
| 4-10-2022 | 1 | Information session with nurses to provide information about the research | A | 1 |
| 4-10-2022 |  | Action research team | B | 1 |
| 14-10-2022 |  | Action research team | C | 1 |
| 2-11-2022 | 2 | Observation | B | 8 |
| 2-11-2022 | 2 | Unit managers | B | 1 |
| 3-11-2022 | 2 | Observation | C | 8 |
| 9-11-2022 |  | Action research team | B | 1 |
| 9-11-2022 | 2 | PhotoVoice | B | 3 |
| 10-11-2022 | 2 | Observation | B | 4 |
| 17-11-2022 | 2 | Observation | B | 4 |
| 17-11-2022 | 2 | Observation | C | 4 |
| 22-11-2022 |  | Action research team | B | 1,50 |
| 22-11-2022 | 2 | Observation | C | 4 |
| 28-11-2022 |  | Action research team | C | 1,50 |
| 1-12-2022 |  | Action research team | A | 1 |
| 9-12-2022 | 2 | PhotoVoice | C | 1 |
| 13-12-2022 |  | Action research team | A | 1,50 |
| 14-12-2022 |  | Action research team | C | 1,50 |
| 16-12-2022 | 2 | PhotoVoice | C | 1 |
| 21-12-2022 |  | Action research team | B | 1,50 |
| 30-12-2022 | 2 | PhotoVoice | C | 1 |
| 4-1-2023 |  | Action research team | B | 1,50 |
| 11-1-2023 |  | Action research team | C | 1,50 |
| 18-1-2023 | 2 | Observation | A | 4 |
| 18-1-2023 |  | Action research team | B | 1,50 |
| 18-1-2023 | 2 | Interview | B | 0,50 |
| 19-1-2023 | 2 | Observation | A | 4 |
| 19-1-2023 |  | Action research team | A | 1,50 |
| 19-1-2023 | 2 | Unit managers | A | 1 |
| 19-1-2023 | 2 | Interview | B | 0,50 |
| 20-1-2023 | 2 | Observation | A | 4 |
| 25-1-2023 | 2 | Unit managers | B | 1 |
| 25-1-2023 |  | Action research team | C | 1 |
| 27-1-2023 | 2 | Observation | A | 8 |
| 30-1-2023 |  | Action research team | A | 1,50 |
| 31-1-2023 | 2 | Interview | B | 0,50 |
| 1-2-2023 | 2 | Interview | B | 0,50 |
| 1-2-2023 | 2 | Interview 06 | C | 0,50 |
| 3-2-2023 | 2 | Interview 09 | C | 0,50 |
| 8-2-2023 |  | Action research team | C | 1,50 |
| 8-2-2023 | 2 | Unit managers | C | 1 |
| 10-2-2023 | 2 | PhotoVoice | A | 1 |
| 10-2-2023 | 2 | Interview 08 | C | 0,50 |
| 17-2-2023 | 2 | PhotoVoice | A | 1 |
| 24-2-2023 |  | Action research team | B | 1 |
| 24-2-2023 | 2 | Interview 07 | C | 0,50 |
| 24-2-2023 | 2 | Interview 10 | C | 0,50 |
| 1-3-2023 |  | Action research team | A | 1,50 |
| 8-3-2023 |  | Action research team | C | 1,50 |
| 15-3-2023 |  | Action research team | A | 1,50 |
| 16-3-2023 |  | Action research team | B | 1 |
| 17-3-2023 | 2 | Interview 11 | A | 0,50 |
| 23-3-2023 | 3 | Team workshop | C | 2,25 |
| 28-3-2023 | 2 | Interview 12 | A | 0,50 |
| 28-3-2023 | 3 | Team workshop | C | 2,25 |
| 29-3-2023 |  | Action research team | B | 1 |
| 30-3-2023 |  | Action research team | A | 1,50 |
| 5-4-2023 |  | Action research team | C | 1,50 |
| 7-4-2023 | 2 | Interview 14 | A | 0,50 |
| 11-4-2023 | 2 | Interview 15 | A | 0,50 |
| 11-4-2023 | 2 | Interview 16 | A | 0,50 |
| 12-4-2023 |  | Action research team | B | 1 |
| 13-4-2023 |  | Action research team | A | 1,50 |
| 18-4-2023 | 2 | Interview 13 | A | 0,50 |
| 21-4-2023 |  | Action research team | C | 1,50 |
| 26-4-2023 |  | Action research team | B | 1 |
| 2-5-2023 |  | Action research team | A | 1,50 |
| 5-5-2023 |  | Action research team | C | 1,50 |
| 8-5-2023 |  | Update about the research project and share the findings and actions with cluster managers |  | 0,5 |
| 19-5-2023 |  | Action research team | C | 1,50 |
| 25-5-2023 |  | Action research team | B | 1 |
| 26-5-2023 | 4 | Evaluation | A | 1 |
| 30-5-2023 |  | Update about the research project and share the findings and actions with cluster managers |  | 0,5 |
| 2-6-2023 |  | Action research team | C | 1,50 |
| 7-6-2023 |  | Action research team | B | 1 |
| 16-6-2023 |  | Action research team | C | 1,50 |
| 20-6-2023 | 4 | Information session with nurses to provide information about the research | A | 1 |
| 30-6-2023 | 3 | (Team) workshop ‘Art of changing’ for action research team nurses and other interested nurses | all | 2,50 |
| 6-7-2023 | 4 | Evaluation | B | 1 |
| 14-7-2023 |  | Action research team | C | 1,50 |
| 25-8-2023 |  | Action research team | C | 1 |
| 21-9-2023 |  | Action research team | C | 1 |
| 10-10-2023 | 4 | Evaluation | C | 1 |

*Notes:* a. (1) pre-orientation (2) orientation (3) planning and testing actions 3; (4) evalations
