# Supplementary material for: Navigating uncertainties for promoting nurse-led changes in work environments: A participatory action research
Source: Int J Nurs Stud Adv. 2024 Nov 12;7:100265. doi: 10.1016/j.ijnsa.2024.100265 (PMC11625307; doi:10.1016/j.ijnsa.2024.100265)
Supplement: Supplementary file 2 [file mmc2.docx]

# Supplementary file 2. Interview guides

Ward C

We have been exploring your nursing work environment. We had two introductory meetings, conducted various observations, and we organized three Photovoice sessions where we reflected on the work environment. From this, several issues have emerged, all with one overarching theme: engagement with and connection to each other during work.

1. Do you recognize this theme? Why or why not?

2. What is your opinion on the engagement within the team? How do you see this in relation to management?

3. What do you personally do to stimulate engagement? What helps or what doesn't help?

4. We noticed that the department has many rules and protocols. What are your experiences with this? Do you think this could be related to engagement?

5. In the PhotoVoice sessions, multiple strategies emerged to enhance engagement, with the buddy system being one of them. Could you elaborate on how this system operates in your ward? Additionally, what are your thoughts on its effectiveness? What aspects of the system do you find effective, and are there any shortcomings?

Ward B

1. What was the last change project you were involved in? How did it go? What challenged or motivated you? What hindered you?

2. What was the trigger for that project and what was its goal?

3. Who initiated the project, and who were responsible for its execution?

4. What do you need in change projects to make it run successfully?

Ward A

1. How do you personally handle problems you encounter during your workday? What helps and what doesn’t help in this regard?

2. In what ways do you engage in quality improvement projects during your work? What challenges or motivates you? What hinders you?

3. Can you mention a quality improvement project where you have played a role yourself? Go through it from A to Z (as reflection), where did you have influence?

4. What do you think is needed within your team to systematically address problems and quality improvement projects?

5. The Photovoice sessions revealed frequent issues or areas for improvement on the ward. If you wish to tackle these, what do you perceive as aspects you can influence? Additionally, what do you view as factors beyond your control?
