# Supplementary material for: Navigating uncertainties for promoting nurse-led changes in work environments: A participatory action research
Source: Int J Nurs Stud Adv. 2024 Nov 12;7:100265. doi: 10.1016/j.ijnsa.2024.100265 (PMC11625307; doi:10.1016/j.ijnsa.2024.100265)
Supplement: Supplementary file 3 [file mmc3.docx]

# Supplementary file 3. Themes, codes, descriptions & representative observations, and quotes

|  | Description | Observations, PhotoVoice & Interviews^a^ | Action Research Team^a^ |
| --- | --- | --- | --- |
| Theme: collaborative working | | |  |
| Good atmosphere | Social interactions and a sense of belonging during nurses’ work | Between the patient tasks that needed to be done, there was a lot of room for informal contact; breaks were coordinated, so they happened together.  Memo of researcher: “Staff is cheerful, team leader is energetic and friendly towards all nurses.” (3/11, Observation, Ward C)  One nurse started her coffee break alone, but she did not mind: *“That way I can respond to patients’ calls for my colleagues, so they don’t have to keep interrupting their break.” (27/1, Observation, Nurse, Ward A)*  *“We have a nice team and nice colleagues, we support each other; this influences the quality of care. Patients also appreciate and experience this” (9/11, PhotoVoice, Nurse, Ward B)*  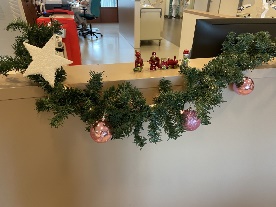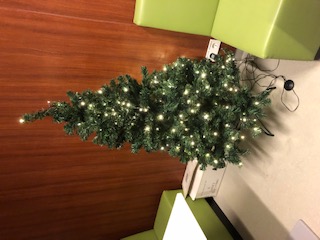  Social interaction was mentioned as a contributing factor to good cooperation.  *“Colleagues are part of your life, your work life. In that sense I think it [involvement] is important. Especially because if you have good mutual contact, patient care will be better.” (10/2, Interview 8, Nurse, Ward C)* |  |
| Feeling valued | Feeling valued by colleagues, managers, patients, and by the organization | Treats from colleagues and patients were appreciated by all ward nurses, positive messages in the coffee room created a pleasant working atmosphere. The fruit, delivered to the ward by the organization, was also appreciated. (General observation)  Nurses linked the photo with sweets and treats to coziness, sweetness, enjoyment, making the break more pleasant, positive energy, being taken seriously, and being seen.  *“Having put in your utmost effort for three weeks straight, appreciation is truly welcomed. Delightful treats then help to turn things around.” (9/11, PhotoVoice, Nurse, Ward B).*  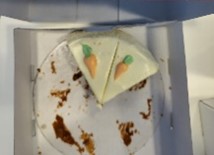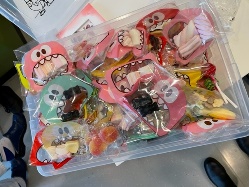  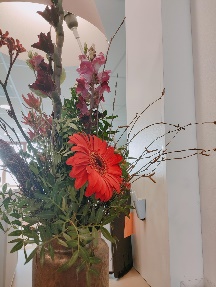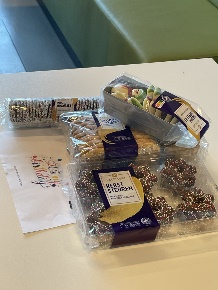 |  |
| Engagement | The level of involvement, interest, and commitment that nurses have towards a task and towards each other. It involves mutual relationships, making and keeping agreements | All teams emphasized the importance of teamwork. However, large teams made it more difficult to connect with each other. Ward C had a very large team; there was not much connection/collaboration between different units within one ward. The mental well-being of colleagues was not always known, and many nurses experienced challenges as it could influence their way of working and interactions. (General observation)  *“Colleagues often do not volunteer if a colleague wants to exchange a shift. Sometimes there is no involvement with each other.” (17/11, Observation, Nurse, Ward C)*  “*Due to the hard work, we don't always have time for each other or even to take care of ourselves.” (16/12, PhotoVoice, Nurse, Ward C).*  *“You don't know what's going on with everyone, because it's a big team. [...] Sometimes there is a get-well card for someone, but you don't know why.” (1/2, Interview 6, Nurse, Ward C)*  It became apparent that commitment of a team to making changes in the workplace can be a challenge.  *“The next day I'm on a different team again. Let someone else figure out the problem.” (16/12, Photovoice, Nurse, Ward C)*  *“There’s a problem, a solution is being sought. That is presented to the team. And then it slowly fades away. […] If you’re going to change something, it’s for a reason. So, then you have to keep it up. I'm also trying to see for myself: does this work? I have sometimes gone back to such a group: This is decided, but I am running into problems here and there.” (18/1, Interview 1, Nurse, Ward B)* | It was a challenge to keep all action research team nurses informed of the progress within the action research team. This required action from both the present and absent action research team nurses during meetings. Minutes and app groups were mostly used for communication. Ward A had a large action research team, which should have had the advantage that there was always someone present. The disadvantage was that people found it difficult to keep each other informed. The action research team in ward C was smaller, and members seemed to keep each other better informed. Yet, in all wards, 'I wasn't there last time, so I don't know exactly...' was heard regularly.  All action research teams had difficulty keeping their appointments within the set time and distributing the tasks properly. It was accepted because everyone seemed to experience the same reasons for this (lack of time, different prioritization, not seeing each other in between, and irregular schedules).  *“Sometimes you are there and other times you are not, then you miss the feeling. If you see the whole group regularly you feel more connected, which creates more energy to tackle the work.” (26/5, Evaluation, action research team nurse, Ward A*)  *"At a crucial moment, the working group did not proceed twice in a row. Then you lose each other for a while." (26/5, Evaluation, action research team nurse, Ward A)*  *"What we particularly struggled with was the irregularity. We were only able to plan the entire group together after 3 months." (26/5, Evaluation, action research team nurse, Ward A)*  *"Make sure that there are one or two people who are always there and the others around them. Two who are in charge, who monitor the red line and keep everyone informed about the activities and progress. Who have an overview." (26/5, Evaluation, action research team nurse, Ward A)* |
| Dividing tasks | Allocating specific responsibilities and assignments among nurses | Ward nurses had a clear task distribution throughout the day and sometimes discussed patient care with each other in between. In some wards, the patient allocation was made the evening before, while in other wards it was done during the handover. While making this allocation, nurses on a ward could also schedule time to work on quality projects. (General observation)  *“Having your own patients provides an experience of more control over the patient. You are responsible for the entire process.” (3/11, Observation, Nurse, Ward C)*  Nurses linked a photo of a white planning board at the ward to maintain an overview of all zones within the ward. Colleagues know whether they need/can offer assistance and what the situation is in the ward. This improves collaboration. (9/11, PhotoVoice, Ward B) | Within one ward, it seemed like there was a discrepancy in task distribution among the action research team nurses. Some nurses were involved, while others did not feel included. In contrast, in another ward, tasks were divided among all action research team members irrespective of attendance during the working group.  *“The attendees took up the tasks and that made the ones that were not present less involved, because they did not know what to do.” (26/5, Evaluation, action research team nurse, Ward A)*  Even when tasks were divided during the action research team meetings, nurses only sometimes accomplished these tasks, and they only sometimes seemed to encourage each other to carry out tasks The exception was on ward C later on in the research project when they started to feel responsible |
| Teaming up | The desire to work (physically) on something together instead of alone, receiving support from colleagues | Ward nurses enjoyed working collaboratively. Buddy systems existed in two wards to stimulate collaboration: conducting double medication checks, distributing breaks, discussing specific details with each other, and supporting each other in the patient tasks. (General observation)  *“I think it makes you more involved with each other. [...] Because if you are really buddies, then you are together more. I would find it more enjoyable to work together more. And safer too, I think. Especially for the young people, that they have someone to lean on.” (24/2, Interview 7, Senior nurse, Ward C)* | Among all action research teams, there was a strong desire to work on something together instead of alone. This became especially visible during literature research: when it was not possible to work on it together, tasks were not carried out.  *"When researching literature, it's nice to have a buddy instead of alone, because there is less experience in this." (26/5, Evaluation, action research team nurse, Ward A)*  Nurses preferred working together physically instead of online to avoid misunderstandings.  Action research team nurses experienced difficulties in communicating with the researchers because it was often online.  *“Organizing online working groups is efficient, but not conducive to experiencing the atmosphere. There is less attention for the human side.” (6/7, Evaluation, Manager, Ward B)* |
| Communication about changes | Involves sharing information regarding any changes within nurses’ work (environment), such as updates, or alterations in procedures, protocols, or the overall workflow. | During handovers, attention was given to mainly patient care related changes or modifications, such as maintaining new score lists or a new method for distributing medication. (General observation)  What researchers thought that contributes to successful change: good communication within the team about the usefulness, necessity, and content of a change, repeating and addressing colleagues, and creating support. Create sufficient time for workgroups.  *“What helps with changes is discussing them during the daily evaluation and continuing to repeat them. 'We agreed on something last month, how is that going, are you succeeding, are you running into any problems?' Instead, every few months there is a group of people in that working group who post something by email and discuss it once or twice.”* (18/1, Interview 1, Nurse, Ward B)  *“It [the change] was also difficult at first, but those people just kept repeating it over and over again, repeating it, repeating it, and at a certain point everyone had it in their reports. You should especially explain to people who see less of the usefulness of the change. If they say things are going well and you show the figures, you can't ignore that.” (*19/1, Interview 2, Nurse, Ward B) | There was mainly contact between the action research team and the rest of the team at specific times, for example, at ward meetings or if something was expected of ward nurses. At other times, it was found difficult to keep everyone informed of what was happening in the action research team. In ward C, towards the end of the project, a more informal (one-on-one or small groups of colleagues during the shift) way of informing colleagues took place. That helped to restore calm to the team that was created after a heated team meeting: the action research team had already made some progress and reflected afterward that perhaps they should have included the group better in the process, but also concluded that the team only woke up at that point.  *"I think we have shown the team that we really want to give our own interpretation to it and that we really do it for the team" (10/10, Evaluation, action research team senior nurse, Ward C)*  *"Perhaps we have done too little in the preliminary phase to bring the team along, to already remove some of that resistance, so to speak" (10/10, Evaluation, Manager, Ward C)*  *"Hey, they are going to seriously change something with this project! And they previously thought, we'll see, we'll provide some input, and we'll see." (10/10, Evaluation, action research team senior nurse, Ward C)* |
| Collaboration with other disciplines | Working cooperatively with professionals from different fields | One team had won an innovation award for a digital planning board for the physicians/nurses’ visits to patients.  *“The board is very useful. This gives you more control over the organization of your day. The patient also knows where he or she stands. We are less dependent on the physician.”* (18/1, Observation, Nurse, Ward A)  Collaboration with and appreciation of the physician was discussed (*"they don't understand what nursing looks like"*), the other way around a physician said: *“Nurses don’t realize all we do.”*. Collaboration with the pharmacy was also not optimal *(“Their staff shortage is made into our problem”)*. But showing leadership to optimize collaboration is not widely shown (9/11, PhotoVoice, Ward B) |  |
| Feedback | Giving feedback, asking questions, and addressing each other about responsibilities | All teams worked well together, but hardly any feedback was given or requested. Colleagues also did not often speak to each other about undesirable behavior, for example, about tasks that had (not) been completed. (General observation)  *“Addressing each other also remains an issue. It's best to tell someone if they have forgotten something. Or you say: maybe you can do it this way next time. That rarely happens. And then they talk about it behind each other's backs. Nobody learns anything from that.” (27/1, Interview 3, Nurse, Ward B)*  *“I think everyone gets along really well and it's a lot of fun. What doesn't really happen is providing structured feedback to each other [...] that could be done differently. That we dare to speak to each other without anyone feeling attacked.” (31/1, Interview 4, Nurse, Ward B)*  *“It is easier to address each other if you know each other better.” (24/2, Interview 10, Nurse, Ward C)* | During the action research team meetings, the nurses did not call each other out on their behavior. It seemed that the integration of reflection or at least structured feedback to achieve improvements collaboratively was difficult for them. (General observation) |
| Theme: developing competence | | |  |
| Skills related to patient care | A diverse set of abilities for providing high-quality patient care | The delivery of patient care is streamlined, and ward nurses knew well what they needed to do. They seek each other out for questions. Clinical lessons on common medical conditions are also regularly organized. (General observation).  *"I know what needs to be done, so I don't worry" (22/11, Observation, Nurse, Ward C).*  *“I've been in the business for a while. I have already seen and experienced a lot. This combination of theoretical knowledge and experience makes me flexible and agile. I adapt and I see what needs to be done.”* (7/4, Interview 14, Nurse, Ward A) |  |
| Skills related to methodical work | The ability to follow established procedures, protocols, and methodologies in patient care | The emphasis on working methodically was different among the three wards. In one ward, all nurses had received a basic LEAN training, while in the other, this integration was less pronounced. Experience with the LEAN method gives structure but can sometimes stand in the way of creativity. The LEAN expertise seems to be easier to apply to practical problems in the ward, not to more abstract issues. (General observation)  *“Where is the methodical nursing process? I feel good patient care starts with a profound nursing process, but I see on our ward there is no proper interpretation of the nursing process.” (17/2, PhotoVoice, Nurse, Ward A)* | The systematic working approach in the project appeared to be new for the action research team nurses. Action research team nurses found it difficult to make a good planning. One action research team was regularly surprised by the tasks that had to be done. This seemed to occur due to a lack of foresight on the part of the action research team nurses. Their expectations of the timeline were amiss, and their planning was not specific enough. Another action research team found the time between meetings (two weeks) too short and felt rushed. Action research team nurses sometimes did not speak to each other in between meetings and only took up tasks shortly before a meeting. The third action research team tended to skip steps due to the need for proper planning. In all wards, nurses did not know that certain tasks were part of the role of an action research team nurse.  *“Above all, planning was not done properly, we did not think ahead well enough. Maybe we didn't know either. We started every meeting from the beginning again.” (26/5, Evaluation, action research team nurse, Ward A)*  Guidance/support to take the right steps was necessary to help nurses better deal with methodical work.  *“I’ve had an afternoon course of project management, but I find it challenging to link that to this project.” (10/10, Evaluation, action research team senior nurse, Ward C)*  *"I am a vocationally trained nurse. Maybe it’s different for a bachelor nurse, but I didn't really have that [project skills] at school, for example. In the beginning I found it quite difficult, so to speak, because I didn't really know what was expected of us and how that [a project] works, because I've never done anything like this before." (10/10, Evaluation, action research team nurse, Ward C)* |
| Skills related to chairmanship | The ability to lead and coordinate projects. To guide and oversee the execution. |  | All action research team nurses encountered the problem of there being no good agreements in the beginning about the division of tasks in the team. It was unclear who the chairman and minute taker were, and no clear working agreements had been made. This caused uncertainty, which meant all action research teams felt the need to arrange this properly. A plan was made for the chairmanship and secretary and what actions were expected from them. In all action research teams, the roles of chairman and minute taker rotated. Not every action research team nurse felt confident in the role of chairman.  *"That [leading projects] is indeed often the responsibility of team managers and then employees certainly go along to projects, but they are mainly there for the content, thinking along and carrying out tasks, but it is different from taking on a project yourself. I don't think that’s the case just for the project group (action research team), but actually for the entire ward. (10/10, Evaluation, Manager, Ward C)*  *"I found it a bit difficult at times what kind of role I had as a senior nurse and I still sometimes think: should I really take that leadership role or that chairman's role or not? Yes, I always have doubts about that. Should I just let someone else have chance to learn it?" (10/10, Evaluation, action research team senior nurse, Ward C).*  *"As a working group we were in the lead, but this [size of the project] was too difficult for us." (26/5, Evaluation, action research team nurse, Ward A)* |
| Skills related to problem identification | Recognizing issues during practice and prioritizing these challenges | During the PhotoVoices, ward nurses took many pictures of issues like disorderly wards, unclean beds, the absence of materials, or disorganized or improperly stored medication. Nurses' reflections on these photos varied from disorganization, chaos, waste, extra time, and irritation to a waste of money.  *“There's a shared responsibility for cleaning up the mess.” (16/12, PhotoVoice, Nurse, Ward C)*  *“Leaving things tidy conveys a sense of calm and control.” (30/12, PhotoVoice, Nurse, Ward C)*  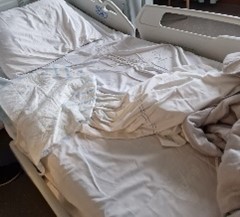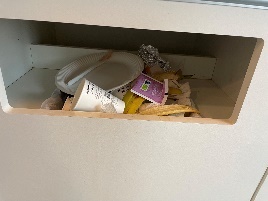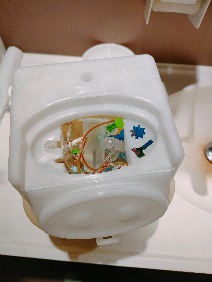  *“I don't enjoy working in an environment where I think: this could be better or more efficient. And because of my training I know how to improve things or how to get things different. That's why I enjoy improving it.” (17/3, Interview 11, Nurse, Ward A)* | In the action research teams, we noticed that nurses were not used to 'zooming out' when a problem occurred and reflecting on it on order to learn from it. |
| Skills related to problem solving and initiating change | The ability to adapt and implement changes within the practice settings | Memo of researcher: ‘Ward nurses are discussing the absence of catheter bags. *"Maybe we should go shopping for them,"* one suggests. Nurses express surprise that this issue hasn't been addressed, as it was the same four days ago. However, none of them indicate an intention to take charge and resolve or investigate the matter.’ (19/1, Observation, Ward A)  A cyclical learning and improvement process seemed to be absent among the ward nurses. We observed empty improvement boards at the wards, and several explanations were given, such as:  *“We do not use it, because this is too much of a hassle” (27/1, Observation, Nurse, Ward A).*  *"If you put it [an issue] up there yourself, you also have to deal with it yourself. Nurses are hesitant to do so because it is perceived as cumbersome. Additionally, the large team makes it challenging to implement changes." (3/11, Observation, Nurse, Ward C).*  *“I want to solve things as quickly and efficiently as possible and not make it bigger than it is. I don't want to take the problem home with me.” (7/4, Interview 14, Nurse, Ward A)*  During the PhotoVoice and interviews we learned that problem solving was mainly based on one's own expertise and opinions and was mainly done for short-term solutions. There was little knowledge in the teams about what is needed to successfully change.  *“Are we going to invest our energy in untangling the wires of ECG machines? I think it's a waste of my time.”* (9/11, PhotoVoice, Nurse, Ward B)  *“The point is that it is solved quickly, not that it is solved with good quality. I think that a structural approach, for something that works in the longer term, often fails to occur.”* (17/3, Interview 11, Nurse, Ward A) | The action research teams had many questions about how to get the team on board with changes. One action research team started investigating which issues could contribute to change management by nurses within their team. Based on literature, observations and interviews, this resulted in an evidence-based overview. This document contained "Ten golden rules for change in nursing teams." The rules emphasized the importance of adding value, team involvement, clear communication, respecting norms and values, feasibility, a systematic approach, extensive communication, leadership support, and continuous attention to the change process.  *"I have come to appreciate the process of change more, instead of the result. Because the process determines success." (6/7, Evaluation, action research team nurse, Ward B)*  Clear communication about planning and timelines from researchers appeared to be important.  *"If you notice from the research that it [the project] is not clear, you can intervene earlier. Because at a certain point the energy is gone and then it no longer gets off the ground and that is a shame." (26/5, Evaluation, Manager, Ward A)* |
| Skills related to research and evidence-based practice (EBP) | The ability to critically analyze and apply research findings to (clinical) decisions | Skills related to research were not yet generally available or used in nursing teams, but more and more attention was paid to it. Deploying EBP skills in the workplace still had challenges.  “*I think that we work very little evidence-based, because some things need to be addressed very quickly. I also noticed this while doing my research. I wanted to approach it completely systematically, researching it carefully first. Yes, the managers were of course not happy that it would take me six months to write a solid plan that I think works. I think that could be better*.” (11/4, Interview 15, Nurse, Ward A)  *“I think it's great that the nursing profession as a whole is developing towards more scientifically substantiated, more EBP. Not every nurse has to be able to do this, take the lead, or be good at it. But they must feel that they are the professionals and that they understand what they do within their own field.”* (17/3, Interview 11, Nurse, Ward A) | Being able to look up and apply literature was not self-evident for most action research team members. Almost all action research team members had difficulty with this, including translating the results into their own context. They found it very challenging to do this independently, but also asked for little or no help from the researchers (despite this being offered).  Most action research team members had little experience and, above all, little confidence in their own research abilities, with the exception of an action research team nurse with a focus area EBP in Ward B.  A vocationally trained nurse searched very hard for her role within the action research team with regard to research.  *“I finally found it in the practical input and translation into practice.” (6/7, Evaluation, action research team nurse, Ward B)* |
| Transitioning from an abstract concept to a concrete outcome | Translating high-level or abstract ideas into tangible, practical results | Ward nurses found it difficult to look creatively and out-of-the-box at structural solutions to problems unless it was very practical (workarounds, which do not solve the underlying problem) (Observations and PhotoVoices all wards)  “*What encourages me is that the work you put into it yields something. I like tackling something in a results-oriented manner and that makes me happy.” (11/4, Interview 15, Nurse, Ward A)* | The switch to making the results of the first part of the research concrete seemed to reveal a difference between the wards. It was suggested that Context-Intervention-Mechanisms-Outcomes were used to describe outcomes. In one ward this did not get off the ground (it was not really understood, little help was asked, and action research team members were very skeptical). In another ward, the Context-Intervention-Mechanisms-Outcomes first seemed to bring structure, but ultimately, they were not developed, and the action research team examined how changes could be initiated through the existing working groups. It provided the necessary structure in the third ward and action was taken accordingly. In the third ward things did not go well at once, but the action research team did not give up and managed to get things as desired.  *“I think it's just difficult for us to get the [problem] into concrete terms. That may also be why they [action research team] find it difficult to decide which steps to take.” (25/1, Conversation researchers with managers, Manager, Ward B)* |
| Learning and development opportunities | Educational opportunities and professional growth activities, learning from the research project | During the Photovoice session on Ward B, it was highlighted as a positive aspect that there are ample learning and development opportunities available on the ward.  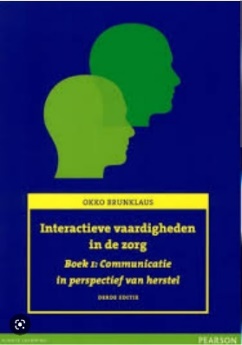  However, not all experiences regarding educational opportunities were positive.  *“I arrange a varied learning program for students and nurses. Where student attendance is good, only a handful of nurses attend, for example, file or case discussions. I miss the feeling that everyone wants to keep learning.” (16/12, PhotoVoice, Nurse educator, Ward C)* | For many action research team members it was the first project they ran by themselves and where they had direct control. They learned from their experiences, and it was a great way to gain experience with projects.  *“I think I have gained more experience.” (26/5, Evaluation, action research team nurse, Ward A)  "When you look back on it like this, you really realize what you have learned" (10/10, Evaluation, action research team nurse, Ward C)* |
| Insecurity about the task | Lack of confidence or uncertainty regarding nurses’ ability to successfully perform a specific task | During a multidisciplinary meeting, the nurses do not sit at the table with the physicians, but on a chair on the 'second row'. A nurse shakes with her hands when she is speaking. Although the physicians are informal and seem to see the nurses as part of the team. (17/11, Observation, Ward B)  *“Some nurses still feel reluctant to say something to a physician, for example if they disagree with something. [...] I think we can really speak out against a physician to stand up for a patient, for example, or to exchange ideas.”* (31/1, Interview 4, Nurse, Ward B) | The action research teams indicated that the responsibility they had been given was a big step compared to what they were used to. All managers had taken a step back and that was not easy for the action research teams. They were used to more guidance, especially at the start. This created uncertainty.  *“As a group we needed more coaching. Who will take us by the hand?” (26/5, Evaluation, action research team nurse, Ward A)*  Self-confidence and positive experiences were needed to handle the responsibility. Ward C was most successful in developing this self-confidence and increasingly taking responsibility. The perseverance of the working group and the attitude of the manager seemed to play an important role in this.  Action research team nurses experienced a lack of clarity about the tasks. As tasks became clearer to them and they felt more confident, there was more preparation.  *“At the beginning, I didn’t really know what to do, but as I got further into the process, I just knew what was expected of me, and did that, and I really enjoyed seeing that I got better and better at it.” (10/10, Evaluation, action research team senior nurse, Ward C)* |
| Theme: accountability | | |  |
| Proactive attitude | Taking initiative and feeling responsible, having a proactive mindset to actively seek solutions | Proactivity and taking responsibility seemed to come easier when it concerned daily patient care than with indirect patient care activities.  *"I always schedule an early shift the day before the medical grand rounds to be adequately prepared. With just the handover and a quick overview, you do not have enough information." (17/11, Observation, Nurse, ward B)*  Ward nurses were able to come up with solutions to problems they experienced, but ideas were quickly dismissed because they did not consider it as their task or responsibility.  *“Before we can use the device, we spend a considerable amount of time untangling the cords. […] We use this device multiple times a day, and a better design would help us use it more effectively.” (9/11, nurse, PhotoVoice, Ward B)*  To bring about change, nurses needed ownership and a sense of urgency and interest in the subject.  *“If they don't see the importance of what needs to be addressed, then I don't think they're going to do it. Then maybe they will do something to please us, For a while, but not for long.” (11/4, Interview 15, Nurse, Ward A)*  *“[I am also working on] medication identification with scanning the patient's wristband, so that you immediately have your two people identification, that change goes a lot better. I think they [the team] just see the need for that more.” (31/1, Interview 4, Nurse, Ward B)*  In the process of improving the working environment, it also turned out that individualism often prevailed in a ward (‘I don't have a problem, so why does something need to change?’) | At the beginning, one action research team took responsibility for scheduling appointments, but not for the project in general. On ward C, responsibility increased during the project. When the action research team received a lot of negative attention after a ward meeting, they did take responsibility for the work they had delivered (and stood behind it!) and did not shift this responsibility to others. (Observation researchers)  action research team members often only started to think about the tasks at hand when the next meeting was almost there, which meant there was no thorough preparation. (Observation researchers)  *“I missed the intrinsic motivation: ‘Yes! We are going to work on this!’” (26/5, Evaluation, Manager, Ward A)*  If tasks were unclear or something did not work out, little responsibility was taken. There was little communication that something did not work out, nor was there any help requested to make it happen. If something was too difficult, it was often not done, for example, during literature research (under the excuse of "no time"). It was said it would be done, but it didn't happen. (Observation researchers)  *"I often thought it was my fault if I didn't understand something. That hindered me from asking." (26/5, Evaluation, action research team nurse, Ward A)* |
| Taking a leading role | Taking responsibilities that go beyond direct patient care | Nurses were used to taking responsibility in daily practice. However, they found it much harder to handle responsibilities beyond direct nursing care.  *“The team disengages and is difficult to reach, but for the patient, they do everything.” (14/12, action research team nurse, Ward C).*  *“I think it is positive when nurses take the lead in changes. We are also at the bedside, and if we have to change things, it would be nice if we determine the bottlenecks ourselves. It can be determined from above, but then it [the solutions] is not always feasible.” (19/1, Interview 2, Nurse, Ward B)*  *"What I mainly saw is that nurses actually indicate that they really want to have a say, but they don't actually know how to do that effectively. They [nurses] want leadership and control, but would like to invest that [responsibility] elsewhere" (10/10, Evaluation, Manager, Ward C)* | Most action research team nurses had a wait-and-see attitude. However, there were differences between nurses. In every action research team, there was someone who naturally had a more leading role. In ward C, taking a leading role seemed to get easier as the group gained more control and ownership of the project. They increasingly succeeded in thinking outside the existing rules and becoming more creative. First version 'utopias' were guided by the limitations of the current situation instead of starting from the desirable situation.  *“Towards the end of the project, I got the idea that we had taken more control ourselves, and I think we learned a lot from that" (10/10, Evaluation, action research team senior nurse, Ward C)*  The research project was perceived as an assignment by the action research team nurses, lacking a sense of ownership and fostering a wait-and-see attitude. While ownership increased on some wards, more initiative was displayed, and action research team nurses took a leading role. However, there was still uncertainty about whether individual ideas would meet expectations, which constrained out-of-the-box thinking, as observed by the researchers.  *“Of course, you [the researchers] come from externally. It actually seems more like you are the ones who are leading the way, so they [action research team] don't take the role. How can you turn that around?” (25/1, Conversation researchers with managers, manager, Ward B)* |
| Sense of influence | The feeling of having a meaningful impact on decisions, policies, patient care, or the research project. | Nurses made many assumptions when it came to possibilities. Often, they did not start change because the expectation was that it would not be successful. (Observation researchers)  To get rid of negative points nurses indicated that they needed good cooperation with other disciplines. Many nurses did not feel they had sufficient influence on identified challenges and issues. *"Things are arranged from higher up."*  *(17/2, Photovoice, Nurse, Ward A)*  *“During the PhotoVoice sessions, I noticed remarks like, "I'm not happy about this, but I have no influence over it." However, I believe that, in some cases, these were matters where nurses could exert influence if they knew which routes to explore or with whom to engage in conversation.* (17/3, Interview 11, Nurse, Ward A)  *“The design of the hospital was simply a choice. So I can say that I want an extra cupboard in the room, but... I won't even start with that. That won't happen anyway.”* (18/1, Interview 1, Nurse, Ward B)  *“Nurses do not determine policy but participate in its execution.” (10/10, Evaluation, action research team nurse, Ward C)* | Initially, two wards experienced a restricted sense of influence, affecting the motivation of action research teams to invest effort. In the ward where processes were already effective, a sense of resignation was observed. Nevertheless, as the sense of influence grew, action research team nurses began considering more opportunities, though this continued to pose a challenge.  *“I’m very quick to say: that’s not possible. So I immediately dismiss everything, without investigating first whether it might be partially possible after all. I learned to look from the perspective of the desired situation. That is of course a completely different way of looking at something. Much more is possible than we thought. That was a real eye-opener for me.” (10/10, Evaluation, action research team senior nurse, Ward C)*  On ward A, numerous assumptions were made without verification, leading to the belief that facilitation was not possible. This often resulted in project delays. The action research team felt that the circle of influence was quite small. The ward meetings were an example of this. These had to be planned, but the assumption was that no time could be made for this.  *“It is impossible to plan a two-hour meeting for the entire team” (30/3, action research team meeting, action research team nurse Ward A)* |
| Dealing with setbacks | Learn from setbacks and adapt to unexpected situations | *“I'm quite flexible about it. If there are changes, I think: we can always try. And if it doesn't catch on, it doesn't catch on. At least we tried. And if it does work, it can only be a good thing.” (19/1, Interview 2, Nurse, Ward B)* | Action research teams dealt with setbacks differently. In ward A, the group quickly came to terms with what was not possible. Some action research team members spoke out quite strongly about impossibilities and set the atmosphere. Others in the group hardly objected. The action research team of ward C excelled in resilience. They had a major setback after the ward meeting, with part of the team turning against them. The action research team reflected on this together and determined that they supported their choices and wanted to continue with them. This even caused strengthened motivation and the connection between the action research team members.  Being able to deal with setbacks was important to make progress in improving the working environment. Overcoming setbacks together could also create a connection.  *"We faced challenging moments because the team was sometimes difficult to bring along, but it is also rewarding when you eventually succeed. […] A learning point for myself was indeed not to focus on those who are negative but rather on those who are willing to embrace the change." (10/10, Evaluation, action research team nurse, Ward C)* |
| Reflection | Thoughtful analysis of experiences, actions, and decisions | Reflection, essential to achieve improvements together, appeared to take up virtually no space in all three wards. Within the teams, reflection was mainly about the division of labor. Nurses were not familiar with reflection methods or 1st-3rd order learning.  “*After an acute situation you notice the adrenaline in the nurses. They talk about the situation with each other. They all ask each other how things are going. Very collegial. But it is an evaluation, not a reflection.” (19/1, Observation, Nurse, Ward A)*  Working days were mainly focused on getting patient care completed. Nurses evaluated their work at the end of the day, but this was not focused on learning together.  *“At the end of the day, there is an evaluation. But no one actually tells it like it is. [...] we all say 'yes, nice job'. Everyone is just done, and we won't go into it anymore. I think we miss out on a lot there.” (1/2, Interview 6, Nurse, Ward C)* | Reflection was not part of the action research team working method, unless there was specific time for this with the researchers. Sometimes reflection took place, such as in ward A when things did not run smoothly (this led to new work agreements), but often the reflection remained superficial, or action research team nurses placed matters outside themselves. At the end of the project, there were reflections with action research team members from each ward, where they critically looked at their own roles and reflected on them. This provided insights into how things could be done differently next time, both personally and for the group.  *“At the beginning we discussed that we would rotate (chairman role ed.), but after that I never really clearly discussed my struggle whether this was a good choice or not.” (10/10, Evaluation, action research team senior nurse, Ward C)*  *“We should have looked more specifically at what was expected of us in the timeline and reviewed the goals from the start (..) so that we could plan on time.” (26/5, Evaluation, action research team nurse, Ward A)*  In ward C, after the ward meeting (where a part of the team presented itself negatively), this working group was able to reflect very well on what had happened, how it affected them personally and what they learned from it. They reflected on what their own actions had done to others and what they thought about it.  *“The team meeting was a turning point. Then we thought, okay, we really have to keep it much closer to the team, but we also really have to play our role in that.” (10/10, Evaluation, action research team senior nurse, Ward C)*  *“It is nice that it works out in the end. When you look back on it like this, you really realize what you have learned.” (10/10, Evaluation, action research team nurse, Ward C)* |
| Attributing problems to external factors | Emphasize that challenges or problems in the work environment are influenced by external circumstances rather than personal shortcomings | When things didn't work out or didn't go well, the first reaction was usually that it was due to someone else or to a process, such as workload, lack of support, or processes of not designed for nursing work. Everyone was annoyed by certain things (such as mess), but no one seemed to take responsibility, and almost no one did anything about it. That caused extra annoyance (kind of a vicious circle). (General observation)  *“We came up with this project to increase quality in the ward. However, we notice that we cannot achieve this due to staffing levels. These are all external factors that simply make it extremely difficult to implement the plan we have.”* (11/4, Interview 15, Nurse, Ward A) | When things were not going well or were not clear, the cause was easily blamed on someone else or something outside the action research team. Having to prioritize care was often given as a reason why tasks had not been addressed.  “*There has been little time to look at this quietly in the past two weeks” (30/3, action research team meeting, action research team member, action research team nurse, Ward A)*  *“No one has ever come to me to ask what we did at RN2Blend.” (26/5, Evaluation, action research team nurse, Ward A)* |
| Complaining behavior | Expressing dissatisfaction about work-related issues | At the PhotoVoice meeting, it was said that there was complaining in order to 'get it out of the system' instead of reflecting to improve or change.  *“There is a “resignation in complaining.” People complain to clear their heads instead of wanting to change something.” (10/2, Photovoice, Nurse, Ward A)* |  |
| Theme: support from manager | | |  |
| Level of support | The extent to which nurses receive guidance, encouragement, and resources from their managers | In interviews, the level of support by managers was discussed.  *“I think they are the biggest incentive to make us feel professional. They certainly play a role, both in organizing days for quality work and in strengthening our professional sense (17/3, Interview 11, Nurse, Ward A)*  *“Changes have also been made in other wards where I have worked, but they were not really encouraged by the managers, and that does happen here. [...] I see it as an important stimulating factor if managers also support a change. They can certainly achieve more with changes than if I were the only one to start.” (19/1, Interview 2, Nurse, Ward B)*  *“When I started my master education, I approached my managers to ask how we could structure or implement it. I asked, "Do you have any use for me?" Well, that was all quite challenging, simply because it's unfamiliar territory. So, I didn't feel much cooperation there, and I still don't always.”* *(17/3, Interview 11, Nurse, Ward A)* | Ward C had made the most progress in enhancing the working environment, largely due to strong managerial support. Conversely, ward A faced significant challenges, receiving less support from managers and not actively seeking it. Consequently, there was limited project development within the group. Wards B and C exhibited growth, especially Ward C. Despite the desired autonomy of the action research teams, a certain level of managerial support appeared to be beneficial and necessary in initiating a project successfully. The distinction between Wards B and C lied in the managers' roles: in B, managers mainly contributed upon request, while in C, the manager actively sought input on needed support and coordinated her role with the action research team and researchers.  *“Because as a manager I was expected not to direct anything, so I didn't do that. That also asked something of me. But at a certain point I intervened and said: I think we now have to provide a little more guidance and give the working group more guidance about their role, the role of the researchers and the expectations.”* *(10/10, Evaluation, Manager, Ward C)*  *“I learned to think in a different way from her [manager], because I think she guided us very well in this.” (10/10, Evaluation, action research team nurse, Ward C)*  *"I have also responded to the action research team members: I don't know everything either but indicate what you need and then I can see where I can provide support. And I'm happy to hear that that's a good thing, that it worked." (10/10, Evaluation, Manager, Ward C)* |
| Degree of visibility | How frequently and actively a manager is present and engaged in the daily activities and interactions within the team | The presence of managers at the start of the day was common and was experienced as positive. This not only made managers visible, but it also helped to emphasize the importance of certain matters. (General observation)  *“At the start of the day, they [the managers] also emphasized: 'scan that medication' [current improvement project]. They are very involved, and I think it stresses the importance.”* (31/1, Interview 4, Nurse, Ward B)  *“The managers are involved in the team, but they don't always show it that way, because they sit in the back in an office. Recently a few [managers] were just in white in the ward, a few times when it was busy, and that benefits the cooperation. And it also benefits the understanding, because they see the need and they respond to it.” (24/2, Interview 10, Nurse, Ward C)* | Managers were very willing to facilitate the action research teams. However, some managers waited for explicit requests for help, which were not always made.  Action research team nurses in ward A sometimes felt unsupported, and there were numerous assumptions about the limitations in facilitating their work. Managers took the instruction to 'let go' very literally and did not interfere with the action research team. When they noticed that things were not going well, they asked the researchers for information, not the action research team. They later indicated that they thought they had fulfilled the assignment well by acting like that, even though it sometimes did not feel quite right. In ward B, additional facilitation was considered when needed, and also discussed with the researchers.  Relying solely on waiting for requests may not be sufficient to offer the necessary support. Proactively offering assistance, verifying understanding by asking clarifying questions, and ensuring that help resources are easily accessible are successful strategies. |
| Mirroring responsibility and ownership | Demonstrating a shared sense of accountability and ownership for tasks and decisions | We have only seen this behavior in ward C by the manager. She regularly reflected on the behavior exhibited by both the action research team and the team. This appeared to help change behavior in a positive way. | There was a clear difference between wards. Mirroring did not happen in ward A, in ward B only minimally towards the action research team and in ward C this happened towards both the action research team and the team. The manager of ward C recognized undesirable or counterproductive behavior during the project and spoke to people about this by mirroring and providing insight into the effect of this behavior. The manager asked active questions about which support was needed. The manager complimented the team, and she provided support about the process by questioning the action research team nurses. How do we get through this? The action research team nurses consciously continued with the project. Continuing when things got difficult brought a lot to the nurses (= leadership, longer term, wanting the best for your employees).  *"From the halfway point onwards, we also had her [the manager] more as a source of information and then we just started sparring with her and you learned things from that." (10/10, Evaluation, action research team nurse, Ward C)* |
| Balancing between providing guidance and allowing autonomy | Offering guidance while also granting autonomy to nurses, ensuring a supportive and independent work environment | *"The more I direct and push, the more they [the team] resist." (30/6, Observation Workshop, Manager, Ward C)* | This was quite a challenge and quest for all managers. Ultimately, there appeared to be a major difference in how this was implemented. Ward A let go of too much, which led to 'swimming' and the lack of requests for help. Ward B let go, but had tried to stay in touch with the action research team about progress and to provide assistance where necessary. There had been limited guidance and more attention had been paid to the researchers. Ward C eventually had the right balance between managing and letting go. The action research team was released on the content, although the feasibility of choices was coordinated with the manager. The team was guided regarding process and behavior. The action research team felt supported by the manager, partly because it stood up for them when (part of) the team turned against the action research team. |

*Notes:* a. date format DD/MM is used
